# Enzymatic synthesis of selenium-containing amphiphilic aliphatic polycarbonate as an oxidation-responsive drug delivery vehicle†

**DOI:** 10.1039/c8ra10282a

**Published:** 2019-02-18

**Authors:** Xian-Ling Yang, Xiu Xing, Jun Li, Yan-Hong Liu, Na Wang, Xiao-Qi Yu

**Affiliations:** Key Laboratory of Green Chemistry & Technology, Ministry of Education, College of Chemistry, Sichuan University Chengdu 610064 P. R. China wnchem@scu.edu.cn xqyu@scu.edu.cn +86 28 85415886 +86 28 85415886

## Abstract

Although functional aliphatic polycarbonates (APCs) have attracted prominent research interest as stimuli-responsive biomaterials, the majority of functional APCs are fabricated by detrimental organometallic catalysts or organo-catalysts. Herein, a facile synthetic strategy based on enzymatic polymerization was developed to construct a selenium-containing amphiphilic aliphatic polycarbonate (mPEG-*b*-CMP_45_). Specifically, the selenium in its backbone framework underwent a hydrophobic–hydrophilic transition upon exposure to the abnormal ROS level of the tumor, thus providing a promising platform for ROS-triggered drug release. This amphiphilic mPEG-*b*-CMP_45_ efficiently encapsulated doxorubicin (DOX) *via* self-assembly in aqueous solution and showed an excellent ability to regulate the release of DOX in response to H_2_O_2_ at biologically relevant concentrations (100 μM). These DOX-loaded nanoparticles could easily be internalized into U87 cells and possess the inherent antitumor properties of DOX, while they exhibited much lower cytotoxicity in normal cells HL-7702. Moreover, in many cases, the introduction of selenium caused high cytotoxicity of the materials, but the cytotoxicity results in HL-7702 cells demonstrated the good biocompatibility of mPEG-*b*-CMP_45_. These collective data suggested the potential use of mPEG-*b*-CMP_45_ as a biocompatible and smart drug delivery vehicle.

## Abstract 2

A facile synthetic strategy based on enzymatic polymerization was developed to construct a ROS-responsive polycarbonate served as biocompatible drug vehicle.

## Graphical abstract

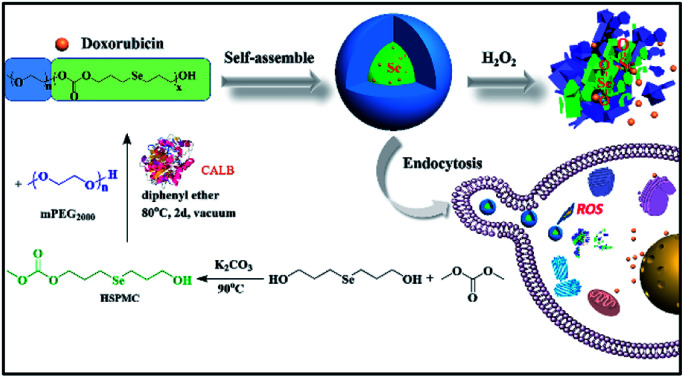

## Introduction

In the field of biomaterials, tremendous efforts have been devoted for the development of stimuli-responsive drug delivery systems that deliver therapeutic agents in a controlled manner. These stimuli could be classified as either endogenous biological parameters (*e.g.*, pH,^1–4^ enzyme,^5,6^ reactive oxygen species (ROS) and reduction conditions^7–16^) or external triggers (*e.g.*, light,^17,18^ ultrasound^19^ and magnetic^20^). Reactive oxygen species (ROS) such as hydrogen peroxide (H_2_O_2_), superoxide, and the hydroxyl radical are one of the most important biological stimuli.^7–9,21–25^ They are extremely common inside the body and act as a double-edged sword. At normal conditions, they serve as key modulators in various physiological processes. However, the undesired elevated ROS is often implicated in many important pathophysiological events, for example, Alzheimer's,^26^ Parkinson's,^27^ atherosclerosis^28^ and some cancer.^29,30^ Based on this difference in normal and pathological tissues, recent studies have proposed a variety of smart polymers to take advantage of the increased ROS level for cancer therapy.

Starting with the development of polypropylene sulfide (PPS) as oxidation-responsive polymeric vesicles in 2004,^31^ various ROS-responsive polymers have been constructed and investigated for the therapeutic purposes. The major classes of reported oxidation-responsive polymers are those containing thioether,^32,33^ thioketal,^34^ arylboronic ester,^35^ aminoacrylate^36^ and peroxalate ester.^37^ Unfortunately, these types of materials usually exhibit unsatisfied response to mild oxidative atmosphere.

To cope with this problem, selenium has been proposed to construct ROS-responsive polymers because of its excellent oxidability. These selenium-containing polymers are more sensitive under mild stimuli conditions than the sulfur containing counterparts, which has inspired researchers to exploit the selenium-containing polymers. Early in 2010, Xu and Zhang successfully designed a selenium-containing polymer PEG–PUSe–PEG and it showed a triggered release of loaded cargos in the presence of 0.1% H_2_O_2_ (v/v), which was more sensitive than sulfur-containing analogue.^38^ And thenceforth, some related studies came into being.^39–45^ Lang and his coworkers have reported a selenium-containing polycarbonate *via* enzymatic ring opening polymerization.^45^ However, the hydrophobicity of the polymer may restrict its application for drug delivery. Notably, there are few studies explored the therapeutic application of selenium-containing polymers, additionally, hardly any of previously reported selenium-containing polymeric drug carriers are sufficiently sensitive to biologically relevant concentrations of H_2_O_2_ (50–100 μM) until now. Hence, it still needs to exploit novel strategy to develop more selenium-containing polymers emphasis on medical potentiality.

Enzymatic polymerization is a powerful tool for synthesizing biopolymers such as polysaccharides,^46^ polyesters,^47^ polyaniline,^48^ aliphatic polycarbonates (APCs)^49^ and so on.^50^ In our continued efforts towards the development of functional polymers *via* enzymatic polymerization, we have explored such as azido-functional,^51^ pH-responsive^52^ and oxidation-responsive polyesters.^53^

Particularly, the development of oxidation-responsive polymers is one of the most active topics in the field of stimuli-responsive polymers. Regrettably, the oxidation-responsive behaviour of our previously reported oxidation-responsive polyester (mPEG-*b*-PTE_20_) showed that mPEG-*b*-PTE_20_ was only sensitive to H_2_O_2_ of high concentration (5%).^53^

**Scheme 1:**
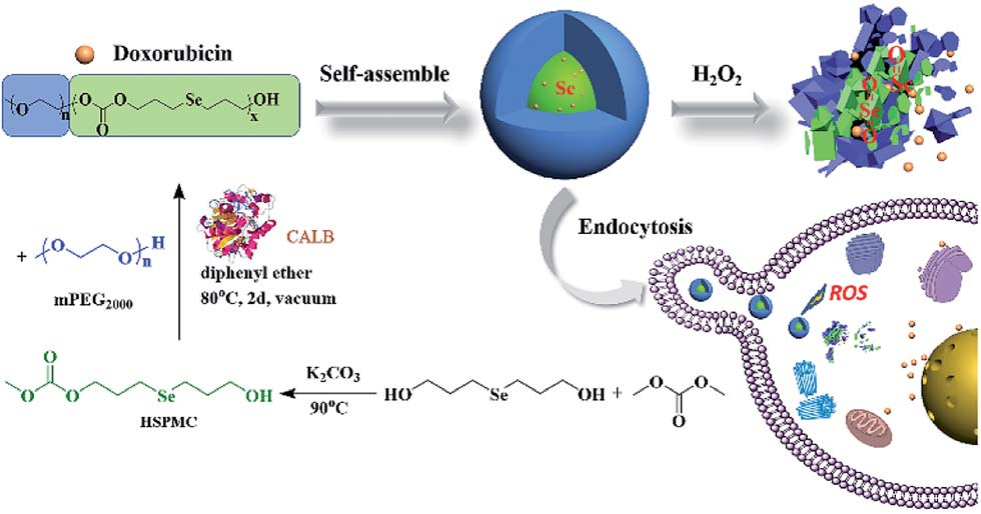
The synthesis of mPEG-*b*-CMP_45_ block copolymers and preparation of DOX-loaded mPEG-*b*-CMP_45_ micelles as oxidation-responsive drug delivery vehicle.

Inspired by our initial work and to further potentiate the ROS-responsive capacity of our materials, herein, we developed a selenium-containing aliphatic polycarbonate (denoted as mPEG-*b*-CMP_45_) using enzymatic polymerization. APCs are one of the most important categories of biocompatible materials on account of the degradation of polycarbonates yield only CO_2_ and diols, which has attracted significant attention of applications in the medical field.^54–57^ In this work, this polycarbonate was endowed with ultrasensitive ROS-responsive capacity with the presence of selenium in its backbone framework. The mPEG was used not only as the chain-terminating agent to obtain this copolymer, but also as a hydrophilic segment endowing the micelles with stealth properties. As shown in Scheme 1, this copolymer was synthesized through a facile process, and it was able to form micelles in aqueous to which the anticancer drug doxorubicin (DOX) can be encapsulated. These DOX-loaded micelles featured selective intracellular drug release and anticancer effect. The results indicated that this selenium-containing polycarbonate would be a promising technology in cancer therapy.

## Experimental details

### Materials

Doxorubicin (hydrochloride, 98%) was obtained from Aladdin Industrial Corporation. And mPEG (*M*_n_ = 2000 Da) was obtained from Sigma-Aldrich and used as received. Novozym 435 (immobilized lipase B from *Candida antarctica*, CALB) was purchased from Novozymes (Bagsvaerd, Denmark) and used as received. Sodium borohydride, selenium powder, 3-bromo-1-propanol, dimethyl carbonate, diphenyl ether, Nile Red (99%) and 30% (v/v) H_2_O_2_ were purchased from commercial suppliers. All solvents and other reagents were analytical grade and used as received. Dulbecco's Modified Eagle Medium (DMEM), 1640 Medium and fetal bovine serum (FBS) were purchased from Invitrogen Corporation. 3-(4,5-Dimethylthiazol-2-yl)-5-(3-carboxymethoxyphenyl)-2-(4-sulfophenyl)-2*H*-tetrazolium (MTS) was purchased from Sigma-Aldrich (St. Louis, MO). HL-7702 and U87 cells were obtained from the Shanghai Institute of Biochemistry and Cell Biology, Chinese Academy of Sciences.

### Characterizations

^1^H and ^13^C NMR spectra were recorded on a Bruker DMX 400 spectrometer with deuterated chloroform (CDCl_3_) as solvents. Electrospray ionization (ESI) mass spectrometry experiments were performed on Shimadzu LCMS-IT-TOF mass spectrometer. The molecular weights and distributions of these copolymers were measured by GPC in THF relative to PS standards on a Waters HPLC system equipped a 2414 refractive index (RI) detector with Waters Styragel® HT3 and HT4 columns in series. The critical micelle concentration (CMC) was determined *via* fluorescence measurements recorded on a Hitachi F-7000 fluorescence spectrometer using Nile Red as probe. Transmission electron microscopy (TEM, Hitachi H-600) studies were performed at an acceleration voltage of 100 kV. Dynamic light scattering (DLS) measurements were performed in aqueous solution using a Zetasizer Nano-ZS90 system from Malvern Instruments with the samples filtered through a 0.45 mm syringe filter prior. Cellular uptake and intracellular drug release study of DOX-loaded micelles was visualized by confocal laser scanning microscopy (CLSM, LSM 780).

### Synthesis of bis(3-hydroxypropyl)selenide

Bis(3-hydroxypropyl)selenide was prepared *via* the previously reported protocol with a few modifications.^58^ Typically, sodium borohydride (0.38 g, 10 mmol) was dissolved in 5 mL of water and Se powder (0.39 g, 5 mmol) was added slowly with magnetic stirring. After the mixture turned to colorless, a solution of 3-bromo-1-propanol (1.4 g, 10 mmol) in 25 mL of anhydrous THF was injected into it. All the procedures were run under Ar flow to prevent oxidation of the selenide ions and selenium-containing products. The reaction was performed at 55 °C for 48 h and the obtained solution was concentrated under vacuum diluted. Subsequently, the solution was extracted with CH_2_Cl_2_ and dried with anhydrous Na_2_SO_4_. Then the product was purified by column chromatography with a 8 : 1 (v/v) mixture of CH_2_Cl_2_ and ethyl acetate as eluent, and pale yellow oil was obtained with a yield of 34%. ^1^H NMR (400 MHz, CDCl_3_, ppm): *δ* 3.69 (t, *J* = 5.8 Hz, 4H, HO*CH*_*2*_), 2.66–2.62 (m, 6H, *HO*CH_2_, HOCH_2_*CH*_*2*_), 1.89 (t, *J* = 6.2 Hz, 4H, Se*CH*_*2*_). ^13^C NMR (100 MHz, CDCl_3_, ppm): *δ* 62.14, 32.89, 20.25; ESI-MS *m*/*z*: calculated 221.0057 for [M + Na]^+^, found 221.0057.

### Synthesis of 3-((3-hydroxypropyl)selanyl)propyl methyl carbonate (HSPMC)

Bis(3-hydroxypropyl)selenide (2.3 g, 12 mmol) was dissolved in 40 mL of dimethyl carbonate, and then potassium carbonate (1.6 g, 12 mmol) was added to catalyze the reaction. After stirring for approximately 6 h at 90 °C, the residual dimethyl carbonate was removed under reduced pressure and the crude product was purified by column chromatography, eluting with 4 : 1 (v/v) petroleum ether: EtOAc. ^1^H NMR (400 MHz, CDCl_3_, ppm): *δ* 4.21 (t, *J* = 6.2 Hz, 4H, *CH*_*2*_OCOOCH_3_), 3.76 (s, 3H CH_2_OCOO*CH*_*3*_), 3.72 (t, *J* = 5.6 Hz, 2H, HO*CH*_*2*_), 2.63 (m, 4H, Se*CH*_*2*_CH_2_), 2.01 (m, 2H, SeCH_2_*CH*_*2*_CH_2_OCOOCH_3_), 1.90 (m, 2H, SeCH_2_*CH*_*2*_CH_2_OH). ^13^C NMR (100 MHz, CDCl_3_, ppm): *δ* 155.70, 67.34, 62.32, 54.76, 32.81, 29.45, 20.32, 19.54; ESI-MS *m*/*z*: calculated 279.0112 for [M + Na]^+^, found 279.0118.

### Lipase-catalyzed synthesis of mPEG-*b*-CMP copolymers

A series of diblock copolymers were synthesized *via* enzymatic polymerization. Initially, the monomer HSPMC and mPEG (*M*_n_ 2000 Da) in various ratios were dissolved in diphenyl ether solvent (200 wt% *vs.* total substrates). And then Novozym 435 (10 wt% *vs.* total substrates) was added to form reaction mixtures. The reactions were carried out under reduced pressure at 80 °C for 48 h. At the end of the reactions, the obtained mixtures were diluted by CH_2_Cl_2_ and were filtered to remove Novozym 435. The obtained crude products were precipitated in *n*-hexane and then washed by a 1 : 3 (v/v) mixed solvent of chloroform and *n*-hexane for three times to get rid of the residual diphenyl ether. The data on copolymer yield, molecular weight is summarized in Table 1.

**Table 1:** Characterization of mPEG-*b*-CMP copolymers

Sample	mPEG/eHSPMC (feed ratio)	Composition^a^ (ratio)	Yield (%)	*M*_w_ b (Da)	*M*_w_/*M*_n_^b^	CAC^c^ (mg L^−1^)	Size^d^ (nm)	PdI^d^
mPEG-*b*-CMP_15_	1 : 12	1 : 15	73	4693	1.77	26.67	102.0 ± 0.9	0.27 ± 0.009
mPEG-*b*-CMP_30_	1 : 25	1 : 30	84	5135	1.83	9.40	140.1 ± 0.5	0.22 ± 0.003
mPEG-*b*-CMP_45_	1 : 35	1 : 45	82	5895	1.58	4.63	150.8 ± 1.2	0.18 ± 0.004

a Determined by ^1^H NMR.

b The molecular weight and *M*_w_/*M*_n_ were determined by GPC against PS standards.

c Calculated by fluorescence measurements using Nile Red as probe.

d Measured by DLS.

e HSPMC represents monomer 3-((3-hydroxypropyl)selanyl)propyl methyl carbonate.

#### mPEG-*b*-CMP_15_

^1^H NMR (400 MHz, CDCl_3_, ppm): *δ* 4.23 (t, *J* = 6.2 Hz, 59H, *CH*_*2*_OCOOCH_3_), 3.65 (m, 185H, O*CH*_*2*_*CH*_*2*_O), 3.38 (s, 3H, *CH*_*3*_OCH_2_CH_2_O), 2.64 (m, 59H, Se*CH*_*2*_CH_2_CH_2_), 2.04 (m, 59H, SeCH_2_*CH*_*2*_CH_2_). ^13^C NMR (100 MHz, CDCl_3_, ppm): *δ* 155.08, 70.57, 67.35, 62.36, 32.88, 29.47, 20.38, 19.68.

#### mPEG-*b*-CMP_30_

^1^H NMR (400 MHz, CDCl_3_, ppm): *δ* 4.23 (t, *J* = 6.4 Hz, 120H, *CH*_*2*_OCOOCH_3_), 3.65 (s, 184H, O*CH*_*2*_*CH*_*2*_O), 3.38 (s, 3H, *CH*_*3*_OCH_2_CH_2_O), 2.64 (m, 120H, Se*CH*_*2*_CH_2_CH_2_), 2.04 (m, 120H, SeCH_2_*CH*_*2*_CH_2_). ^13^C NMR (100 MHz, CDCl_3_, ppm): *δ* 155.08, 70.57, 67.35, 62.35, 32.88, 29.47, 20.38, 19.68.

#### mPEG-*b*-CMP_45_

^1^H NMR (400 MHz, CDCl_3_, ppm): *δ* 4.22 (t, *J* = 6.2 Hz, 180H, *CH*_*2*_OCOOCH_3_), 3.65 (s, 185H, O*CH*_*2*_*CH*_*2*_O), 3.38 (s, 3H, *CH*_*3*_OCH_2_CH_2_O), 2.63 (m, 180H, Se*CH*_*2*_CH_2_CH_2_), 2.03 (m, 180H, SeCH_2_*CH*_*2*_CH_2_). ^13^C NMR (100 MHz, CDCl_3_, ppm): *δ* 155.07, 70.57, 67.35, 62.34, 32.89, 29.47, 20.38, 19.68.

### Determination of critical aggregate concentrations (CAC)

The CAC was determined using Nile Red as a fluorescence probe. Briefly, various concentrations of polymers (ranged from 1.0 × 10^−6^ mg mL^−1^ to 1.0 mg mL^−1^) were added to the sample bottles, and the concentration of Nile Red was fixed at 1.0 × 10^−6^ mol L^−1^. The fluorescence spectra were recorded from 575 nm to 750 nm at an excitation wavelength of 560 nm using a F-7000 fluorescence spectrometer (HITACHI) at room temperature. The intensity of the intensity values of fluorescence emission *I*_617_ (the maximum emission wavelength of Nile red) was analyzed as a function of the logarithm of the copolymer concentration. The CAC values were determined by the cross-point of the linear regression lines.

### Preparation and characterization of blank and DOX-loaded micelles

The copolymers were directly dissolved in deionized water to form the blank micelles. And the DOX-loaded micelles were fabricated using a bulk solvent evaporation method. In detail, the copolymer mPEG-*b*-CMP_45_ (50 mg) with DOX (5.0 mg) was dissolved in 5 mL of THF. Then, 20 mL of DI water was added dropwise into the dispersion, followed by stirred overnight to evaporate THF. The resulting mixtures were filtered by 0.45 μm syringe filters and used for dynamic light scattering (DLS) and transmission electron microscopy (TEM) measurements.

### Determination the concentration of DOX in DOX-loaded micelles solution

1 mL of DOX-loaded micelle aqueous solution was dissolved in 9 mL of DMF, and quantification was performed with a calibration curve of DOX in DMF/water (9 : 1) by fluorescence spectrophotometer with excitation wavelength at 500 nm.

### Oxidation-responsive properties of mPEG-*b*-CMP_45_ micelles

To study the size and morphology change of mPEG-*b*-CMP_45_ micelles after oxidation, the copolymer was dissolved in water and mixed with a 10 mM H_2_O_2_ aqueous solution, and the final concentrations of the copolymer and H_2_O_2_ were 340 mg L^−1^ and 100 μM, respectively. The reaction was performed at 37 °C for 24 h under 100 rpm stirring and the obtained solutions were directly detected by TEM.

### *In vitro* drug release assay

The oxidation-responsive drug release was investigated. 2 mL of 10 times diluent DOX-loaded micellar solution was poured in a dialysis tube (cutoff *M*_w_ 1000) that was placed into 30 mL of 100 μM H_2_O_2_ solution under constant orbital shaking (100 rpm) at 37 °C. At different time intervals, aliquot samples of 1 mL volume were withdrawn from the dialysis tube for fluorescence measurements and dropped back after measurements. The amount of released DOX was evaluated by comparing the remaining fluorescence with the original fluorescence.

### Cellular uptake and intracellular drug release study

The internalization and distribution of DOX-loaded micelles was visualized by confocal laser scanning microscopy (CLSM). U87 glioblastoma cells were seeded on confocal microscope dishes in 1 mL of complete DMEM and incubated overnight at 37 °C in a humidified atmosphere containing 5% CO_2_. Subsequently, the medium was replaced with another 1 mL of fresh DMEM containing DOX-loaded micelles and the cells cultured for another specified time intervals at 37 °C. Then the cells were fixed with 5% paraformaldehyde and the nuclei were stained with Hoechst 33342. Thereafter, fluorescence was examined by CLSM (LSM 780) under excitation at 405 nm for Hoechst 33342 and 488 nm for DOX.

### *In vitro* cytotoxicity study

U87 and HL-7702 cells were utilized to evaluate the cytotoxicity of blank copolymer micelles and DOX-loaded copolymer micelles using MTS assay. A density of 1.0 × 10^4^ cells per well were seeded on the 96-well plates in DMEM medium containing 9% (v/v) FBS. After 24 h of incubation, 100 μL of the medium containing different concentrations of blank micelles and DOX-loaded micelles were added. And the cells were cultured for an additional 24 h, the cell viability was quantified by MTS assay.

## Results and discussion

### Synthesis and characterization

**Fig. 1:**
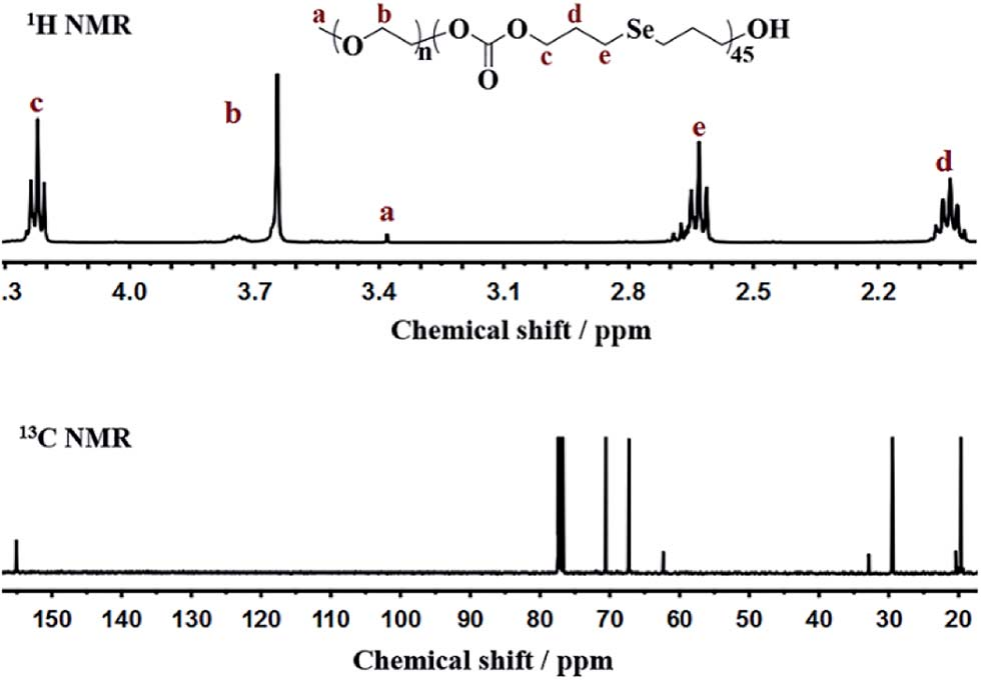
NMR spectra of mPEG-*b*-CMP_45_ in CDCl_3_.

Bis(3-hydroxypropyl)selenide was synthesized according reported literature, and the monomer HSPMC was obtained by the reaction between it and dimethyl carbonate in the presence of potassium carbonate. As shown in Scheme 1, the monomer was polymerized under reduced pressure using mPEG (*M*_n_ = 2000 Da) as a chain-terminating agent and Novozym 435 as a catalyst, which is commercial and easy to separate. The use of reduced pressure during the polymerization could efficiently remove the methanol byproduct to increase the molecular weights of the products. As shown in Table 1, a series of reactions with different monomer feed ratios were performed to afford block copolymer mPEG-*b*-CMP in good yield. The chemical structures of the mPEG-*b*-CMP block copolymers were confirmed by ^1^H NMR and ^13^C NMR. As shown in Fig. 1, the ^1^H NMR spectra of mPEG-*b*-CMP_45_ showed that the resonance of the SeCH_2_CH_2_*CH*_*2*_OH for HSPMC at 3.69 ppm completely disappeared and the characteristic signals of both mPEG and HSPMC units were clearly detected in the ^1^H NMR and ^13^C NMR spectra of the mPEG-*b*-CMP_45_, which indicated the successfully synthesis of mPEG-*b*-CMP_45_ diblock copolymers. The detailed assignments of protons for other copolymers were shown in Fig. S2.† And the degree of polymerization (DP) of selenium-containing units in the copolymers were calculated by comparing the integral ratio of the peaks at 3.38 ppm (terminal methyl peak of mPEG) with 2.04 ppm (SeCH_2_*CH*_*2*_). Additionally, the composition of mPEG-*b*-CMH_45_ was also analyzed and confirmed by ^1^H NMR and ^13^C NMR (Fig. 1). The GPC chromatograms indicated the copolymer mPEG-*b*-CMP have unimodal molecular weight distributions. And the *M*_w_ values of the copolymers kept increasing on the addition of HSPMC monomers, which was in good agreement with hypothesis. The results suggested that the structure and the molecular weights of the copolymers could be well controlled by adjusting the monomer HSPMC ratio.

### Self-assembly behavior of mPEG-*b*-CMP copolymer

**Fig. 2:**
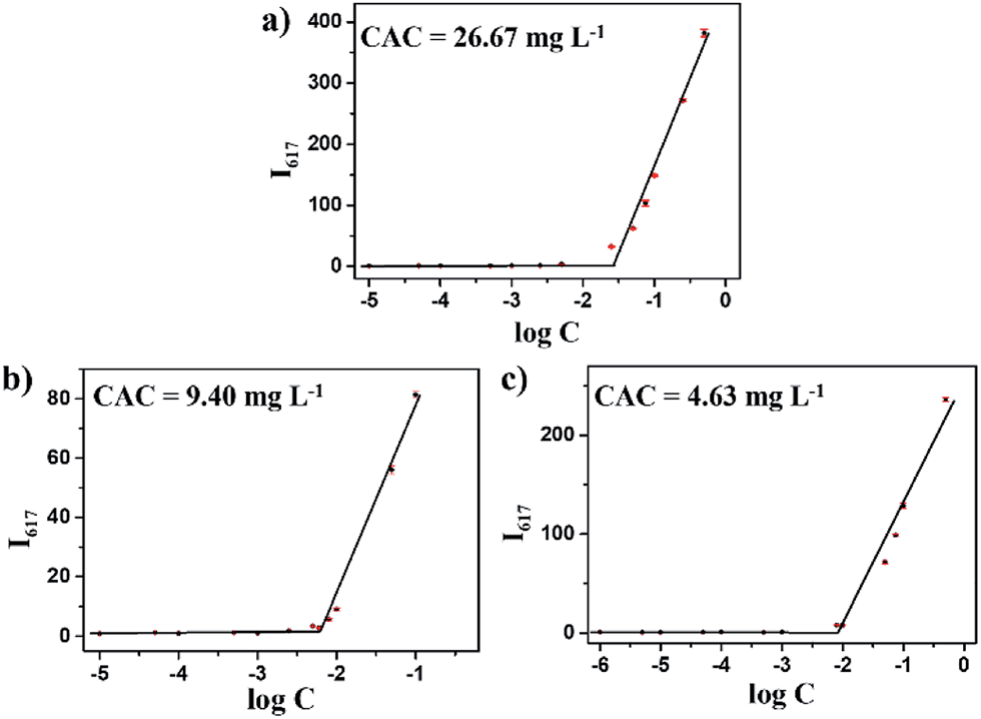
The CAC determinations of (a) mPEG-*b*-CMP_15_, (b) mPEG-*b*-CMP_30_, (c) mPEG-*b*-CMP_45_. The standard deviation for each data point was averaged over three samples (*n* = 3).

The amphipathic copolymers were designed because the architecture enables formation of nanoparticles with core–shell structures in aqueous solution. To study the self-assembly behavior, the critical aggregate concentrations (CAC) were measured using Nile Red as a fluorescence probe (Fig. 2). As shown in Table 1, the CAC values of mPEG-*b*-CMP_15_, mPEG-*b*-CMP_30_ and mPEG-*b*-CMP_45_ decreased from 26.67 to 4.63 mg L^−1^, indicating the copolymers could self-assemble into nanoparticles through hydrophobic interaction and the self-assembly could be readily controlled by adjusting the ratio of hydrophobic segment.

**Fig. 3:**
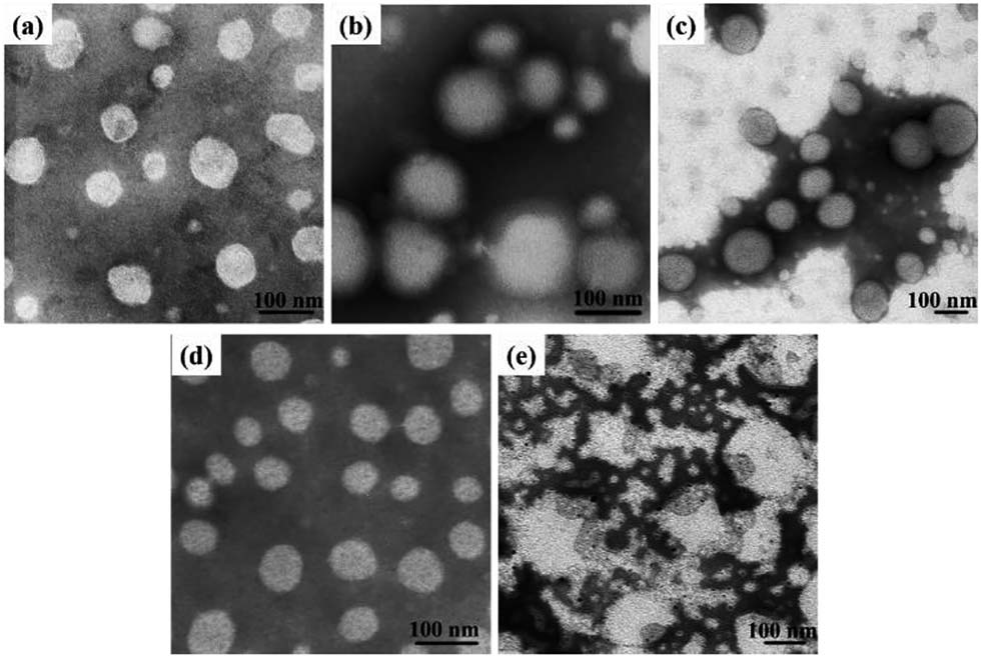
TEM images of (a) copolymer mPEG-*b*-CMP_15_ micelles (68 nm), (b) copolymer mPEG-*b*-CMP_30_ micelles (101 nm), (c) copolymer mPEG-*b*-CMP_45_ micelles (120 nm); and TEM images of mPEG-*b*-CMP_45_ after reaction in (d) water and (e) 100 μM H_2_O_2_ aqueous solution after 24 h.

Dissolving the mPEG-*b*-CMP in deionized water resulted in the formation of self-assembly nanoparticles. Transmission electron microscopy (TEM) and dynamic light scattering (DLS) were utilized to investigate the properties of these nanoparticles. TEM revealed that the obtained self-assemblies were all spherical micelles, and the mean diameters of mPEG-*b*-CMP_15_, mPEG-*b*-CMP_30_ and mPEG-*b*-CMP_45_ were increased from 68 nm to 120 nm (Fig. 3). The results directly proved the mPEG-*b*-CMP copolymers were capable of forming nano-sized micelles *via* a self-assembling process and an increased ratio of the hydrophobic segments effectively enhanced the hydrophobic core. And the DLS results further demonstrated these micelles had an average diameter less than 200 nm (Table 1). The size of nanoparticle plays pivotal role in the applications of intracellular drug delivery because small-sized nanoparticles (<200 nm) tend to maintain a minimal renal excretion, low-level uptake by the reticuloendothelial system (RES), and effective enhanced permeation and retention (EPR) effect for passive tumour targeting. Fortunately, the data indicated these mPEG-*b*-CMP micelles might be appropriate for drug delivery.

**Fig. 4:**
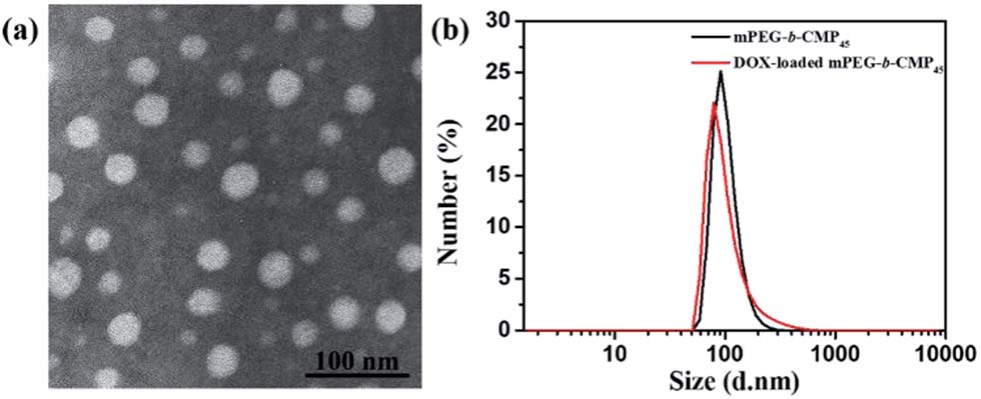
(a) TEM image of DOX-loaded mPEG-*b*-CMP_45_ micelles and (b) DLS results for before and after DOX-loaded in mPEG-*b*-CMP_45_ copolymer.

Based on the self-assembly behavior of mPEG-*b*-CMP, it is possible to encapsulate some guest molecules into the mPEG-*b*-CMP micelles. And we chose mPEG-*b*-CMP_45_ as model because of its feasible water solubility and CAC. Doxorubicin (DOX) as an anticancer drug was selected due to its fluorescence when incorporated into a hydrophobic environment but non-fluorescence while released into a polar environment. DOX-loaded micelles were formed through the process mentioned before, and the final loading capacity of Dox is 60.38 mg L^−1^. As shown in Fig. 4, the TEM and DLS results confirmed that the drug loading did not have significant effect on the morphologies of these micelles, which still had a uniform size distribution and maintained the monodispersed spherical particles.

### Oxidation-responsive properties of mPEG-*b*-CMP_45_ micelles

It has reported that selenium-containing molecules show sensitive oxidation responsiveness, because the hydrophobic selenide groups transform into the hydrophilic selenoxide or selenone groups in the hypoxia environment.^38^ Thus, to assess the ROS sensitivity, mPEG-*b*-CMP_45_ was dissolved in 100 μM H_2_O_2_ to mimic the tumor microenvironment and DI water was utilized as control. As shown in Fig. 3, after exposure in 100 μM H_2_O_2_, spherical micelles were significantly dissociated whereas they remained stable under DI water condition. The results provide the proof of the oxidation-responsiveness of mPEG-*b*-CMP_45_ in the tumor microenvironment and the discernment of tumor microenvironment implied that it could be applied as ROS-triggered drug carrier. Compared with our previously reported sulfur-containing analogue mPEG-*b*-PTE_20_,^51^ the ROS-sensitivity of mPEG-*b*-CMP_45_ has been greatly improved.

### *In vitro* oxidation-triggered drug release

**Fig. 5:**
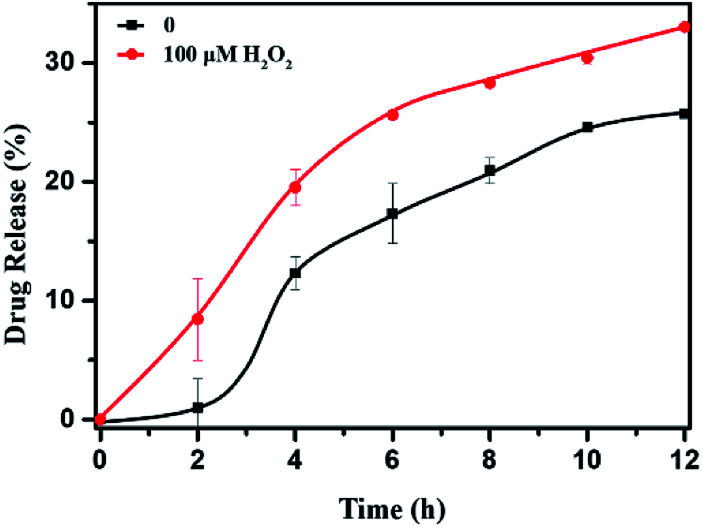
*In vitro* DOX release profiles of DOX-loaded micelles with 100 μM H_2_O_2_. The standard deviation for each data point was averaged over three samples (*n* = 3).

The DOX-loaded mPEG-*b*-CMP_45_ micelles were incubated with 100 μM H_2_O_2_ aqueous solution at 37 °C to mimic the presence of an accelerated pathophysiologic oxidative microenvironment. As given in Fig. 5, the release rate of DOX from mPEG-*b*-CMP_45_ micelles was significantly increased within the first 2 h when 100 μM H_2_O_2_ was added. The DOX was released from mPEG-*b*-CMP_45_ micelles in a sustained manner during a 12 h period. The data demonstrated that the hydrophobic DOX molecules could be shielded in the hydrophobic selenide-containing cores of mPEG-*b*-CMP_45_ micelles, and successfully released under hypoxic conditions, primarily owing to dissociation of the micelles *via* a transformation of the hydrophobic selenide to hydrophilic selenoxide or selenone. The ROS-responsive released manner was also evidenced by the elevated amount of released DOX. And this would enable the ROS-triggered release of DOX within the abnormal environments in cancer cells.

### Cellular uptake and intracellular drug release

**Fig. 6:**
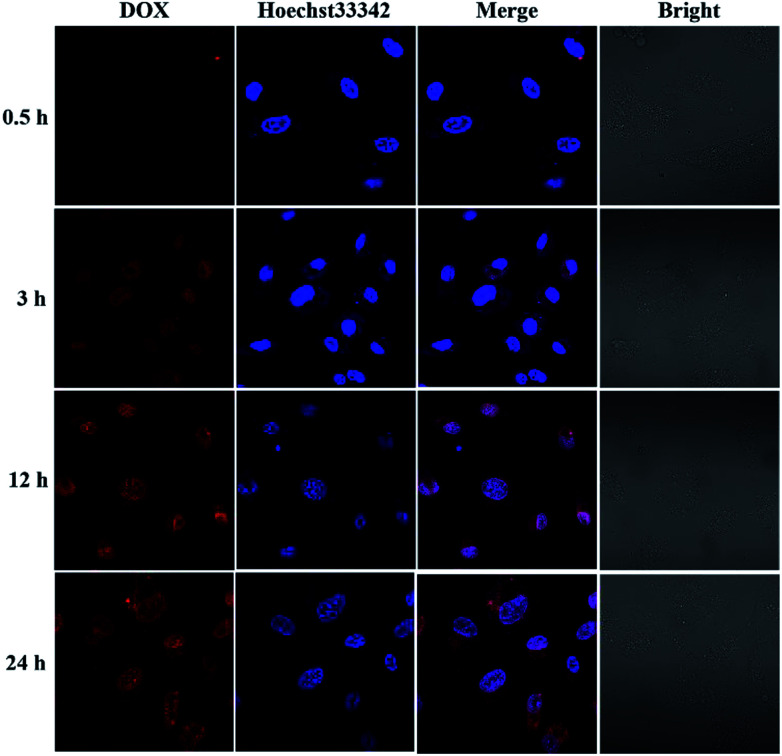
Confocal laser scanning microscopy (CLSM) images of U87 cells after treatment with DOX-loaded mPEG-*b*-CMP_45_ with different duration. DOX dosage was 0.5 μg mL^−1^.

To investigate the intracellular ROS-triggered release, we conducted *in vitro* studies. Remarkably, efficient internalization through cellular uptake ensures subsequent efficiency of cytotoxicity against cancer cells. And there was previous research indicates that the maximum size of nanoparticles for cell uptake is around 50 nm,^59^ so the DOX-loaded mPEG-*b*-CMP_45_ micelles were ideal to be utilized in drug delivery system. To verify the internalization and distribution of DOX-loaded mPEG-*b*-CMP_45_ micelles, CLSM was used to monitor the micelles after incubation with U87 glioblastoma cells. After 0.5 h incubation, the micelles were efficiently internalized by cells according to the fluorescence of DOX in cells. As shown in Fig. 6, with increasing incubation period, the micelles were internalized more and gradually DOX was released from the micelles and distributed intensively in the cytoplasm and marginally in the nucleus. In pace with the incubation time rising to 12 h, the cellular uptake rate has reached maximum and the entering of released DOX into the nucleus was plentiful. The observed results suggested the fast internalization of DOX-loaded mPEG-*b*-CMP_45_ micelles and the DOX was released form mPEG-*b*-CMP_45_ in a rapid process after the micelles were endocytosed into the cells. Therefore, we conjectured the released drug was expected to induce the apoptosis of cancer cells.

### *In vitro* cytotoxicity assay

**Fig. 7:**
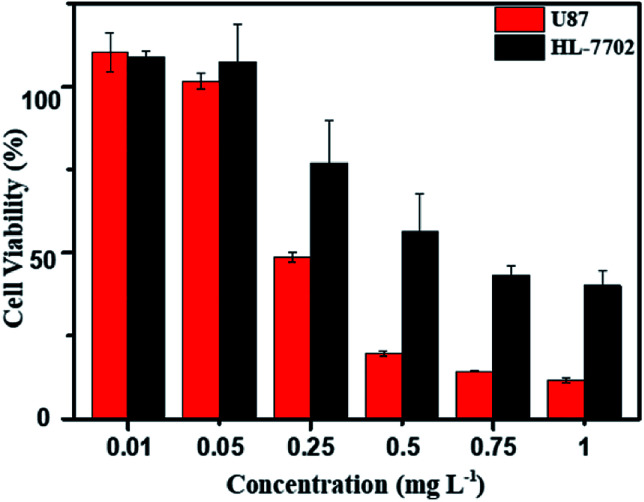
MTS assay of DOX-loaded mPEG-*b*-CMP_45_ micelles in U87 and HL-7702 after incubation for 24 h. The blank cells were used as control and the standard deviation for each data point was averaged over three samples (*n* = 3).

As above mentioned, the DOX-loaded mPEG-*b*-CMP_45_ micelles were confirmed to be successfully internalized by cells and released the loaded DOX. To evaluate the efficiency of intracellular drug release from micelles and achieve its intrinsic anticancer effect, the anticancer abilities of DOX-loaded mPEG-*b*-CMP_45_ micelles was measured *in vitro* against U87 cells using MTS assay. Cells were cultured 24 h for adherence and then incubated with DOX-loaded mPEG-*b*-CMP_45_ micelles at different concentrations from 1 × 10^−3^ to 1 mg L^−1^ for 24 h. For comparison, MTS assay of DOX-loaded micelles in normal human liver cells (HL-7702 cells) was also carried out. As shown in Fig. 7, the DOX-loaded nanoparticles in HL-7702 cells exhibited lower cytotoxicity than that in U87 cells following 24 h of incubation. The dose of loaded DOX in mPEG-*b*-CMP_45_ micelles required for IC_50_ against HL-7702 cells is 0.71 mg L^−1^, which is five times than U87 cells (0.13 mg L^−1^). It is reasonable that the DOX-loaded mPEG-*b*-CMP_45_ micelles exhibited the faster drug released rate in the cancer cells and showed the targeting toxicity on account of the abnormal microenvironment of cancer cells. To further confirm whether the cytotoxicity is induced by the DOX released from the micelles, the viability of the U87 cells incubated with the blank micelles was measured in the same conditions. As shown in Fig. S3,† it was found that no cell death was induced by the mPEG-*b*-CMP_45_ polymer at the measured concentrations up to 100 mg L^−1^, while the maximum dose of mPEG-*b*-CMP_45_ in foregoing anticancer experiments was less than 50 mg L^−1^. This data confirmed the DOX released form micelles possessed inherent antitumor properties. Interestingly, the viability of normal human liver cells (HL-7702 cells) was higher than U87 cells incubated with mPEG-*b*-CMP_45_ at higher concentration. The results proved the excellent biocompatibility of the copolymer that might be attributed to the structure of the copolymer, which was constructed by biocompatible mPEG and polycarbonates. The results showed clearly that the copolymer possessed desirable traits for biomedical applications.

## Conclusion

In this study, a novel oxidation-responsive polycarbonate was successfully constructed with the stimuli-responsive segments in its backbone framework *via* facile enzymic polymerization. In aqueous solution at room temperature, this copolymer formed micellar nanoparticles *via* self-assembly. It was found that this smart polymer was able to act as a drug delivery vehicle and undergo a controlled release of loaded DOX in a mild oxidation environment. From the cell studies, it was clear that the DOX-loaded copolymer micelles were internalized rapidly and exhibited anticancer activity. We expect that the newly designed oxidation-responsive polycarbonate may open up a new window for more effective drug delivery carriers.

## Conflicts of interest

There are no conflicts to declare.

## Supplementary Material

RA-009-C8RA10282A-s001
