# Supplementary material for: Enzymatic synthesis of selenium-containing amphiphilic aliphatic polycarbonate as an oxidation-responsive drug delivery vehicle
Source: RSC Adv. 2019 Feb 18;9(11):6003–10. doi: 10.1039/c8ra10282a (PMC9060885; doi:10.1039/c8ra10282a)
Supplement: RA-009-C8RA10282A-s001 [file RA-009-C8RA10282A-s001.pdf]

Electronic Supplementary Material (ESI) for RSC Advances.
This journal is © The Royal Society of Chemistry 2019

Supplementary Information

Enzymatic synthesis of aliphatic polycarbonate as oxidation-responsive drug delivery vehicle

Xian-Ling Yang, Xiu Xing, Jun Li, Yan-Hong Liu, Na Wang* and Xiao-Qi Yu*

Key Laboratory of Green Chemistry & Technology, Ministry of Education, College of Chemistry, Sichuan University, Chengdu 610064, P. R. China

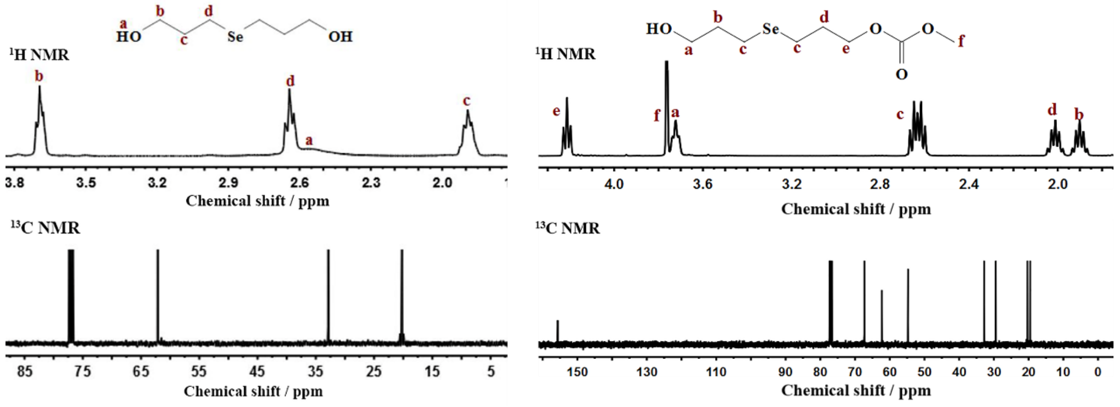

Fig. S1 NMR spectra of (a) bis(3-hydroxypropyl) selenide, (b) 3-((3-hydroxypropyl)selanyl)propyl methyl carbonate in $CDCl_3$.

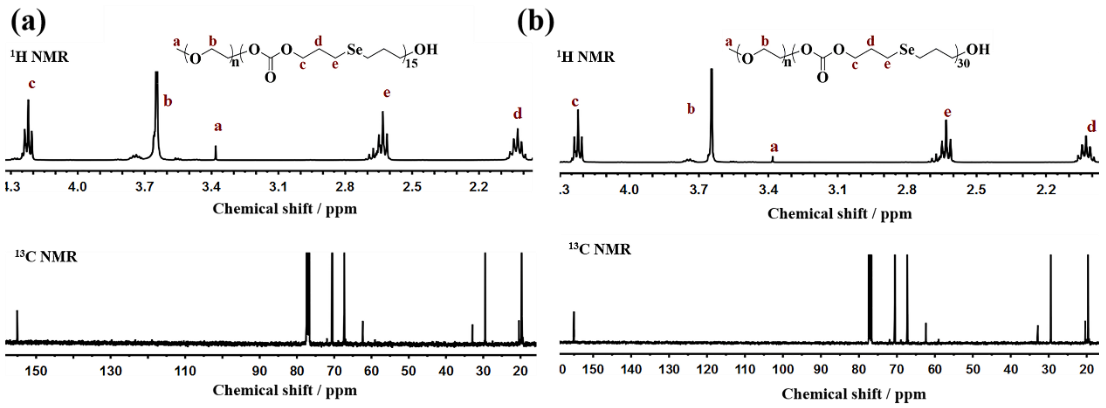

Fig. S2 NMR spectra of (a) mPEG-*b*-CMP_{15}, (b) mPEG-*b*-CMP_{30} in $CDCl_3$.

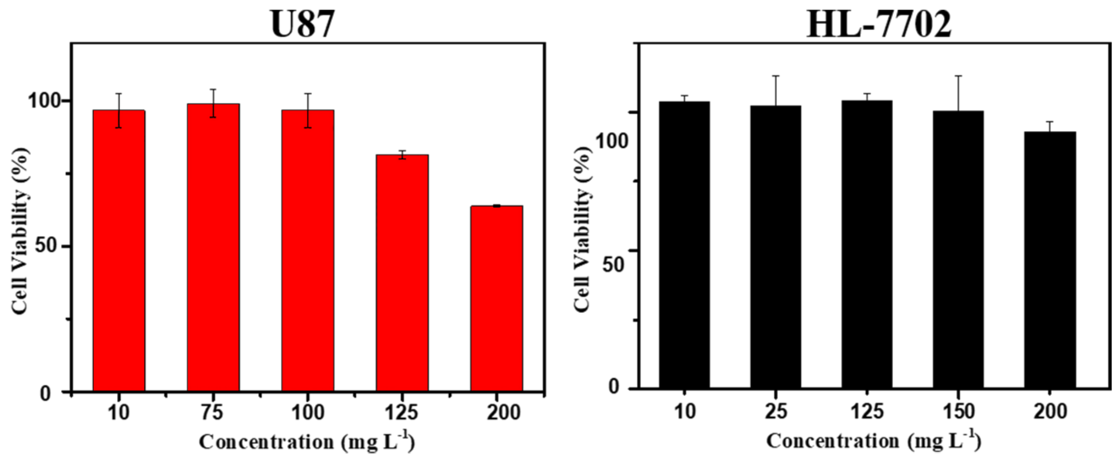

Fig. S3 MTS assay of mPEG-*b*-CMP$_{45}$ in U87 and HL-7702 after incubation for 24 h. The blank cells were used as control and the standard deviation for each data point was averaged over three samples (n = 3).
